# Upper thermal limits differ among and within component species in a tritrophic host-parasitoid-hyperparasitoid system

**DOI:** 10.1371/journal.pone.0198803

**Published:** 2018-06-12

**Authors:** Salvatore J. Agosta, Kanchan A. Joshi, Karen M. Kester

**Affiliations:** 1 Center for Environmental Studies, Virginia Commonwealth University, Richmond, Virginia, United States of America; 2 Department of Biology, Virginia Commonwealth University, Richmond, Virginia, United States of America; CSIRO, AUSTRALIA

## Abstract

Understanding how climate change affects host-parasite systems and predicting the consequences for ecosystems, economies, and human health has emerged as an important task for science and society. Some basic insight into this complex problem can be gained by comparing the thermal physiology of interacting host and parasite species. In this study, we compared upper thermal tolerance among three component species in a natural host-parasitoid-hyperparasitoid system from Virginia, USA. To assess the ecological relevance of our results, we also examined a record of maximum daily air temperatures collected near the study site in the last 124 years. We found that the caterpillar host *Manduca sexta* had a critical thermal maximum (CT_max_) about 4°C higher than the parasitic wasp, *Cotesia congregata*, and the hyperparasitic wasp, *Conura* sp., had a CT_max_ about 6°C higher than its host, *C*. *congregata*. We also found significant differences in CT_max_ among instars and between parasitized and non-parasitized *M*. *sexta*. The highest maximum daily air temperature recorded near the study in the last 124 years was 42°C, which equals the average CT_max_ of one species (*C*. *congregata*) but is several degrees lower than the average CT_max_ of the other two species (*M*. *sexta*, *Conura* sp.) in this study. Our results combined with other studies suggest that significant differences in thermal performance within and among interacting host and parasite species are common in nature and that climate change may be largely disruptive to these systems with responses that are highly variable and complex.

## Introduction

Of the many dimensions to climate change, predicting the response of host-parasite systems to warming has received considerable attention (e.g. [1–14]). Parasitism—broadly defined as including traditional parasites, many plant-feeding insects, parasitoids, and pathogens—is perhaps the most common mode of life on the planet involving interactions among a huge number and diversity of organisms [15–18]. In the terrestrial realm, one common type of host-parasite system is the interaction between herbivorous caterpillars (larval Lepidoptera) and their wasp (Hymenoptera) and fly (Diptera) parasitoids. Unlike typical parasites, parasitoids normally kill their host after larval development [19]. Many host-parasitoid systems also involve a third level of *hyper*-parasitism, with hyperparasitoids that parasitize the original host’s parasitoids.

Predicting how host-parasite systems respond to climate change, and how this may impact ecosystems, economies and human health, is a deeply complex problem [1–3, 9, 12, 14, 17, 18, 20]. At its most basic level, the problem can begin to be dissected by comparing the thermal requirements of component species to infer how changes in temperature may affect the system [1, 3, 6–8]. If, for example, hosts and parasites have similar “thermal windows” for performance, then warming may affect them similarly, and the synchrony of the system (e.g., timing of host and parasite development) may be, to some extent, preserved (e.g. [8, 14]). Alternatively, if hosts and parasites have sufficiently different thermal windows, warming may affect them differently, with the potential to disrupt synchrony and impact population dynamics and stability [10, 21–23]. Furthermore, thermal windows can vary *within* component species, which adds additional layers of complexity to the problem. For example, thermal tolerance is known to vary with age and ontogeny [24–27] and parasitization or infection status [28–32]. To the extent such age-, stage-, and state-dependent variation in thermal windows is important for describing host-parasite systems, modelling and predicting their responses to temperature changes (e.g. [4, 11]) will be all the more difficult.

In this study, we address two basic questions about the thermal biology of host-parasite systems. First, does upper thermal tolerance differ among component species in a natural host-parasitoid-hyperparasitoid (H-P-HP) system? Second, within hosts, does upper thermal tolerance differ with larval stage (instar) and parasitization status? To our knowledge, this is the first study to test for and demonstrate such differences within a natural H-P-HP system. To assess the ecological relevance of our results and the potential implications for climate change, we also examine the frequency of maximum daily air temperatures recorded near the study site in the last 124 years.

## Materials and methods

### Study system

The herbivore, *Manduca sexta* (L.) (“tobacco hornworm”) (Lepidoptera: Sphingidae), is a specialist on solanaceous plants. Its range extends from Southern Ontario to Florida and south to Argentina [33]. Within North America, it is abundant along the Gulf Coast through the Mississippi Valley and the East Coast up to Maryland and New Jersey [34]. This species is a major defoliator of cultivated tobacco, particularly late in the growing season when populations are large [35]. In Virginia, this species has two to three generations per year. *Manduca sexta* serves as an important model organism for insect physiology and development (e.g. [36, 37]) and, in interaction with *Cotesia congregata*, as a model system for host-parasite interactions and insect immunology (e.g. [38, 39]) as well as tritrophic interactions (e.g. [40, 41]).

The parasitic wasp, *Cotesia congregata* (Say) (Hymenoptera: Braconidae), is a gregarious koinobiont and the only hymenopterous parasitoid of *M*. *sexta*; reportedly, it also attacks ~14 other sphingid species in North America [42]. Typically, this species attacks 2^nd^ through early 4^th^ instar caterpillars and can oviposit up to 300 eggs at one time [43]. Wasp larvae undergo two larval instars inside the host and molt to the final 3^rd^ instar while egressing from the host by perforating the cuticle with their mandibles. Larvae then spin and undergo pupation within individual silken cocoons that remain attached to the caterpillar host [43].

The hyperparasitic wasp, a member of the *Conura* (formerly *Spilochalcis*) species complex (possibly, *Conura side* [Walker 1843] [Hymenoptera: Chalcididae]), is one of four common species reported to attack the pre-pupal or pupal stages of *C*. *congregata* on *M*. *sexta*, as well as other braconids and ichnuemonids [44, 45]. This hyperparasitoid was by far the most abundant species at the study site at the time insects were collected for this study.

### Field collection and laboratory rearing

*Manduca sexta* eggs and caterpillars, with and without *C*. *congregata* cocoons, were collected on 5 separate days during July-August 2015 at a privately-owned organic farm in Nottoway County near Blackstone, VA (37.01499, -78.0359), where tobacco has been grown for the past 100 years. Permission to work at the study site was granted by the owner, Mr. Johnny Bledsoe. Approximately 25–50 eggs and caterpillars were collected per field trip and then transported to the laboratory and held at ambient conditions (22 ± 2°C, 30–50% RH). These *M*. *sexta* eggs and caterpillars, and *C*. *congregata* pupae, were the source of all individuals measured in this study, including the hyperparasitoid. Since female *M*. *sexta* lay eggs singly across multiple plants over a period of weeks, and since collections were made on different days over a period of a month, we assume that the stock of field-collected caterpillars and by extension parasitoid wasps used for our experiments represented a random sample of the populations at the study site during the collection period and that the probability of collecting related eggs or caterpillars was low.

To obtain non-parasitized 3^rd^-5^th^ instar caterpillars for the experiment, *M*. *sexta* eggs were hatched in small plastic boxes (~15–20 eggs per box) lined with paper towels and provided with fresh tobacco leaf to feed neonate caterpillars. At the 2^nd^ instar, caterpillars were transferred to individual small plastic cups and provided with fresh tobacco leaf each day. Tobacco leaves were collected from the same field site at which caterpillars were collected and stored in a refrigerator to maintain freshness.

To obtain adult parasitoids and hyperparasitoids, caterpillars with and without egressed *C*. *congregata*, were collected from the field. Caterpillars without cocoons were held in plastic shoe boxes (5–12 caterpillars per box) and provided with fresh tobacco leaf each day. When parasitoid egression was observed, caterpillars were moved to individual plastic cups. Likewise, caterpillars with *C*. *congregata* cocoons at collection were held in individual plastic cups. Each brood of emerged parasitoids, which may be the progeny of more than one female wasp, were then transferred to plastic boxes and provisioned with a wet sponge and honey agar. Wasps used in the experiments were selected haphazardly from a total of 15 broods. Cocoons that did not yield *C*. *congregata* were transferred to individual gelatin capsules (Capsuline, # 00) and held at room temperature until emergence of hyperparasitoids. The hyperparasitoid (*Conura* sp.) deposits a single egg in the prepupa of the primary parasitoid (*C*. *congregata*); in the field, a single brood of C. congregata is typically parasitized by multiple individual hyperparasitoids.

Finally, to obtain parasitized caterpillars to compare with non-parasitized caterpillars, individual 1-day old *C*. *congregata* females without prior ovipositional experience were presented with a 3^rd^ instar day 1 caterpillar reared from field collected eggs and allowed a single oviposition. As for non-parasitized caterpillars, parasitized caterpillars were held in individual plastic cups and fed on tobacco leaf until reaching the desired developmental stage for this study.

### Upper thermal tolerance

To quantify upper thermal tolerance, we measured the critical thermal maximum (CT_max_) as defined by the onset of muscular spasms, which indicates a loss of voluntary muscular control [46, 47]. Like most previous studies, we used the dynamic ramping method to estimate CT_max_ [46, 48–51]. This method involves heating an organism at a constant rate until a predefined endpoint, such as the onset of muscular spasms, is observed. Lighton and Turner [48] demonstrated the physiological basis for this method in insects by showing that the point at which the onset of spasms is observed during a temperature ramp is the same as when ants lose the ability to control respiration, presumably due to a loss of spiracle control.

Following previous studies, we used a ramping rate of 0.25°C min^-1^ for all estimates of CT_max_, which is generally considered an adequate rate for equilibration of body temperature with changes in water/air temperature in small ectotherms ([48–51]; and see below). CT_max_ was measured for individual 1-day old (1) non-parasitized and parasitized 3^rd^, 4^th^, and 5^th^ instar *M*. *sexta*; (2) adult *C*. *congregata*; and (3) adult *Conura* sp. To begin CT_max_ trials, individual caterpillars or wasps were placed inside a small glass vial and completely submerged inside a water bath (Huber CC 118A with Pilot One). We used a start temperature of 30°C which is well within the normal physiological range of these species. Prior to ramping, the organisms were held at constant 30°C for 15 minutes to allow body temperature to equilibrate with the temperature of the water bath and air inside the vial.

To observe the onset of spasms, each trial was recorded with a video camera (Sony HDR SR-11) positioned to focus on the organism inside the glass vial during the temperature ramp. Recordings were analyzed using Windows Live Movie Maker software; the water bath temperature when the first muscular spasm was observed was recorded as CT_max_. Videos of each observation (N = 67) are available upon request from the authors. The final, corrected data after calibration (see below) used for analyses in this paper are given as supplementary information (S1 Appendix).

### Calibration of CT_max_ measurements

The above method for estimating CT_max_ assumes the temperature of the water bath (T_water_) at time *t* during the thermal ramp, which was measured, is equal to the air temperature (T_air_) and body temperature of the organism (T_body_) inside the glass vial at time *t*, which were not measured. However, due to thermal inertia, the potential for a lag between T_body_ and T_water_ or T_air_ increases with increasing body size. At the ramping rate of 0.25°C min^-1^, T_body_ of very small ectotherms like the wasps and 3^rd^ instar caterpillars in this study rapidly equilibrates to changes in T_water_ and T_air_ producing little to no lag [48–51]. However, for larger ectotherms like the 4^th^ and 5^th^ instar caterpillars in this study, lags become likely, which can give misleading results especially when comparing organisms of different sizes.

To address this issue, we calibrated our experiment with two ramping trials to test the assumption that T_water_ = T_air_ = T_body_ using a 4^th^ and 5^th^ instar *M*. *sexta* caterpillar, chosen because they represent the two largest size classes in the experiment. Ramps began at 35°C and ended at 50°C at a rate of 0.25°C min^-1^. Prior to ramping, T_body_ was given sufficient time to equilibrate with T_water_ and T_air_. At the onset of ramping, all three temperatures were recorded simultaneously every 2 min until the end of the ramp for n = 30 paired observations. The internal thermometer of the water bath measured T_water_. To measure T_air_, a bare tip T-type thermocouple probe (Cooper-Atkins model 39138-T) was inserted inside a glass vial by punching a hole through the plastic cap and sealing it with silicone to keep out water. The same technique was used to measure T_body_ except a thinner 26-gauge T-type thermocouple (Physitemp Instruments model W-TW-26) was used with the exposed tip inserted through the body wall and into the body core of a frozen-then-thawed caterpillar. Both thermocouples measuring T_air_ and T_body_, each in separate vials, were connected to the same digital thermometer (Amprobe model TMD-52). To the extent that significant lags were detected (i.e., T_water_ ≠ T_air_ ≠ T_body_), these data were used to calculate correction factors to provide better estimates of CT_max_ based on T_body,_ rather than T_water_.

### Maximum air temperatures

To compare with CT_max_, a record of daily maximum air temperatures near the Blackstone, VA study site was obtained from NOAA’s National Centers for Environmental Information (NCEI) from the Global Historical Climate Network-Daily Summaries (GHCN-Daily) database [52]. These data are publicly available upon request from NCEI. The subset we obtained [53] spans the past 124 years (01-Jan-1893 to 19-Mar-2017; N = 103,159 days excluding 1,465 days with missing data) from the greater Richmond, VA area, which is the nearest network of GHCN-Daily stations and about 72 km northeast of the study site.

### Statistical analysis

CT_max_ data approximated a normal distribution based on normal quantile plots and variances were homogeneous among species (Brown-Forsythe test, p > 0.05). A one-way ANOVA was used to test for differences in CT_max_ among species including non-parasitized 3^rd^-5^th^ *M*. *sexta* caterpillars, adult *C*. *congregata* wasps, and adult *Conura* sp. wasps. A two-way ANOVA was used to test for differences within the host *M*. *sexta* including the effects of instar, parasitization status (parasitized vs. non-parasitized), and their interaction.

All analyses were conducted in JMP Pro version 11.1.1. All tests were considered significant at p < 0.05. All means are reported with ± 1 standard error.

## Results

### Body size of component species

The average wet mass of non-parasitized caterpillars used in the experiment was: 3rd instar = 0.080 ± 0.003 g (n = 7), 4th instar = 0.293 ± 0.042 g (n = 8), 5th instar = 2.251 ± 0.202 g (n = 8). The weights of adult *C*. *congregata* (n = 16) and *Conura sp*. (n = 10) wasps used in the experiment were not measured. Both species are similar in body length (~2–4 mm) and much smaller than the caterpillar host. In a laboratory colony of *C*. *congregata* maintained by one of the authors (KMK), the average wet mass in a sample of 1-day old adult males (n = 10) and females (n = 10) was 0.0002 g.

### Calibration of CT_max_ measurements

Simultaneous measurement of T_water_, T_air_, and T_body_ during two separate ramping trials demonstrated that T_body_ closely matched T_air_ in the 4^th^ instar but was consistently about 1.0°C lower in the 5^th^ instar (S2 Appendix). In addition, both data sets showed that T_air_ was consistently about 0.5°C lower than T_water_ (S2 Appendix). Therefore, we subtracted 0.5°C from all values (all caterpillars and wasps) and a further 1.0°C from 5^th^ instar caterpillar values to obtain the final estimates of CT_max_.

### Differences among trophic levels

**Fig 1:**
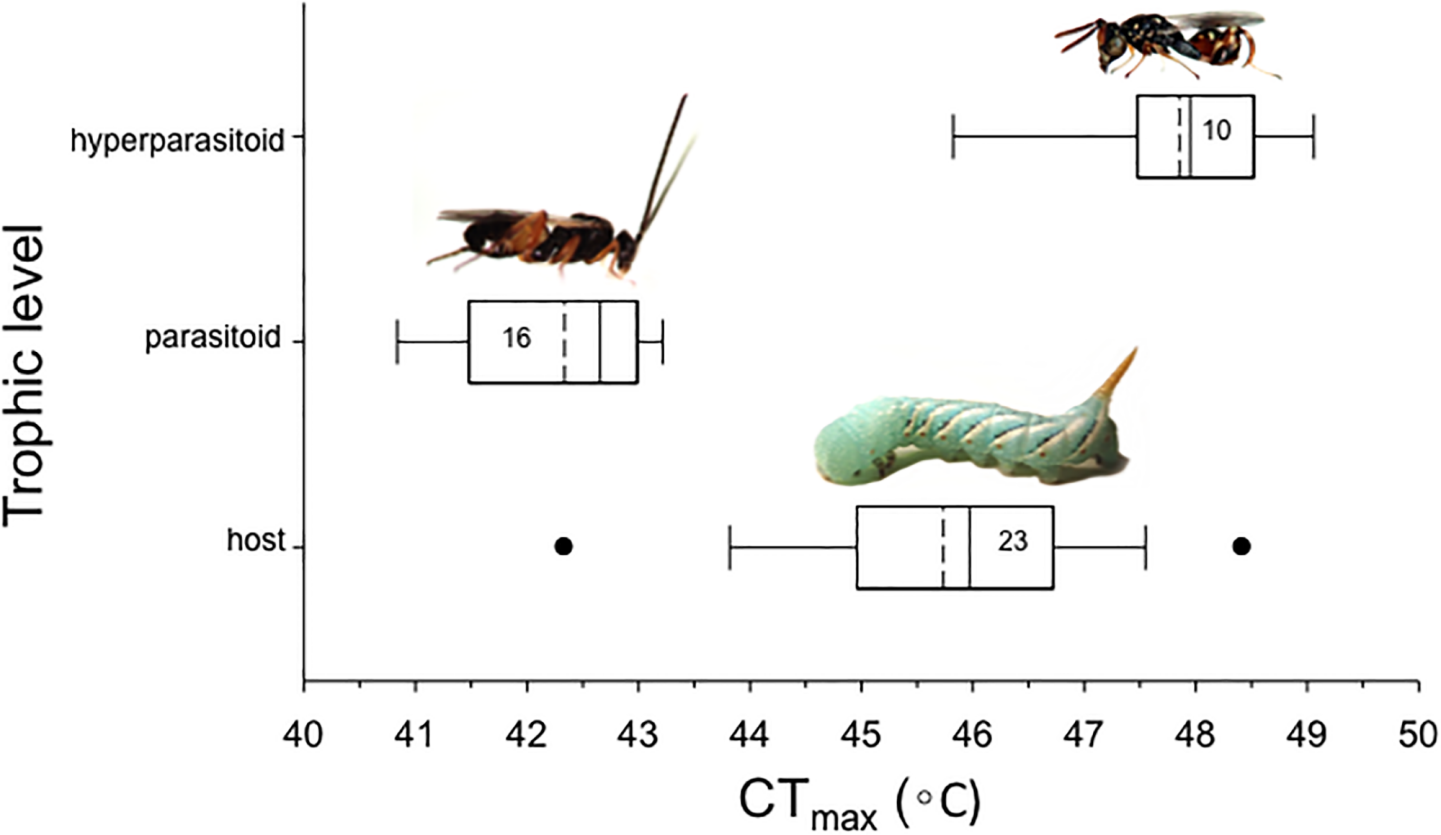
Box-plots of variation in upper thermal tolerance (critical thermal maximum, CT_max_) in component species of a tri-trophic system involving a caterpillar host (*Manduca sexta*), a wasp parasitoid (*Cotesia congregata*), and a wasp hyperparasitoid (*Conura* sp.). Dashed lines = mean; solid lines = median; points = 5^th^/95^th^ percentile outliers. Values inside boxes represent sample size (number of individuals). The means were different among all three species (One-way ANOVA with Tukey HSD post-hoc test; all P’s < 0.05). Photo credits: Justin Bredlau.

There were significant differences in CT_max_ among trophic levels (F = 70.03, d.f. = 2, 46, p < 0.0001; Fig 1). Tukey’s HSD post-hoc test indicated the hyperparasitoid *Conura sp*. had the highest CT_max_ (47.9 ± 0.3°C), the parasitoid *C*. *congregata* had the lowest (42.3 ± 0.2°C), and the host *M*. *sexta* was intermediate (45.7 ± 0.3°C) between the two (Fig 1).

### Differences within the host *M*. *sexta*

**Fig 2:**
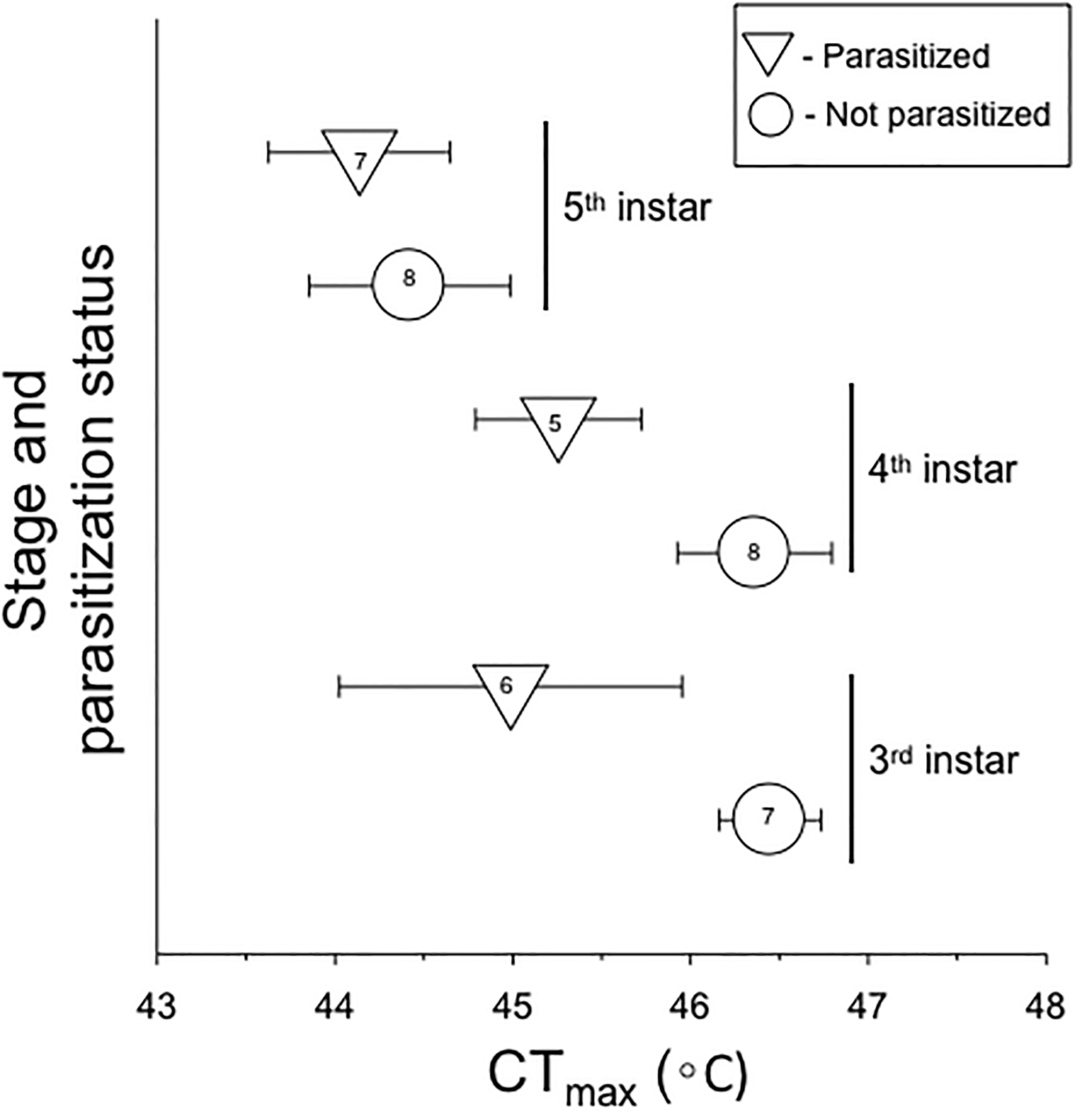
Variation in upper thermal tolerance (critical thermal maximum, CT_max_) within the caterpillar host *Manduca sexta* in relation to ontogenetic stage (instar) and parasitism by the wasp *Cotesia congregata*. Data points represent the mean ± 1 standard error. Values inside data points represent sample size (number of individuals). The main effects of instar and parasitism were both significant (Two-way ANOVA; Table 1).

There were significant differences in CT_max_ within the host *M*. *sexta* (Table 1, Fig 2). A two-way ANOVA found significant main effects of both instar and parasitism on CT_max_, but no significant interaction (Table 1). There was no difference in CT_max_ between 3^rd^ and 4^th^ instars, but it was about 1.5°C lower in 5^th^ instars (Tukey’s HSD post-hoc test; Fig 2). In addition, parasitized caterpillars had a CT_max_ approximately 1.0°C lower than non-parasitized caterpillars (Student’s t post-hoc test; Fig 2).

**Table 1:** Effects of ontogenetic stage (instar) and parasitism by the wasp *Cotesia congregata* on upper thermal tolerance (critical thermal maximum, CT_max_) of *Manduca sexta* caterpillars.

Source	d.f.	Sums of squares	F-ratio	P-value
Instar	2	19.79	4.88	0.0135
Parasitism	1	9.40	4.64	0.0383
Instar x parasitism	2	2.26	0.56	0.5783
Error	35	104.46		

### Maximum daily air temperatures

**Fig 3:**
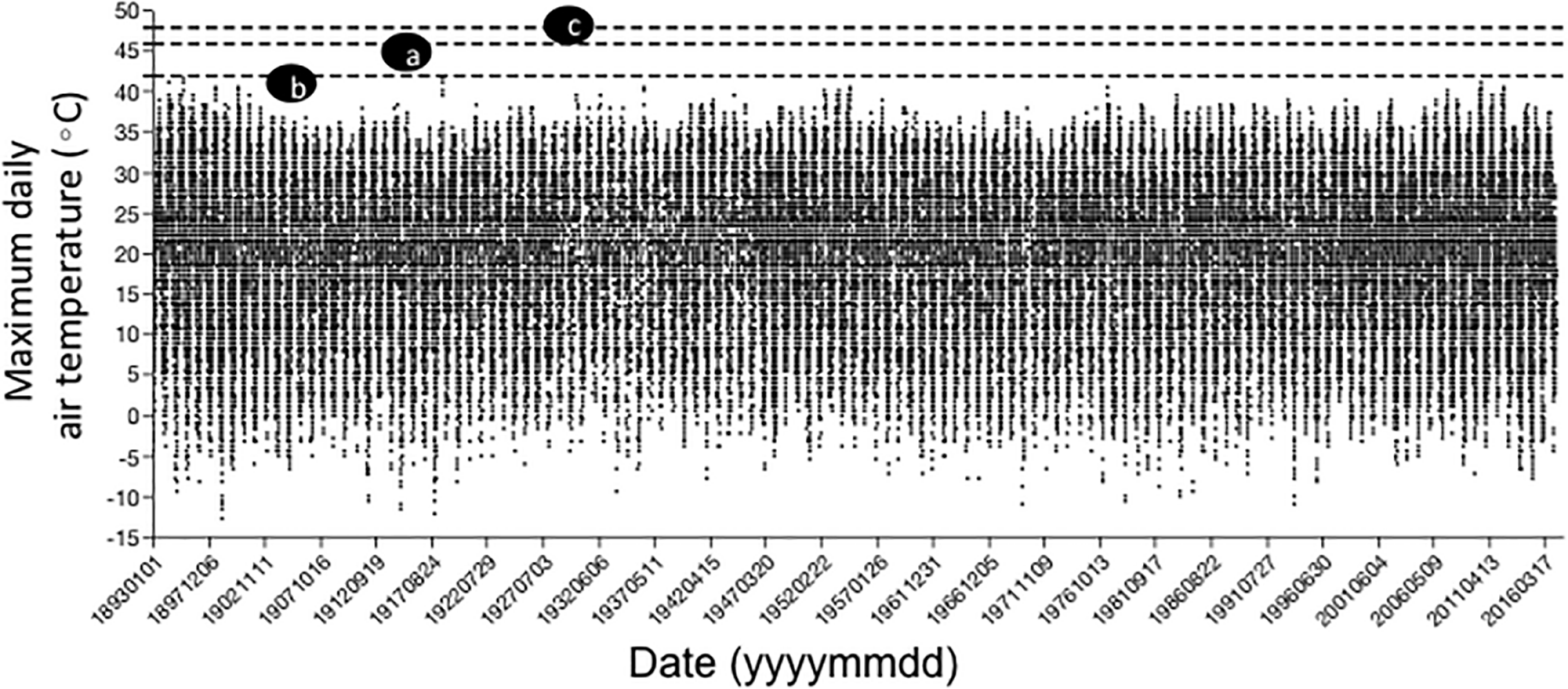
Maximum daily air temperatures recorded near the study site in the past 124 years compared with the average critical thermal maximum (CT_max_) of component H-P-HP species measured in this study. Dashed lines: a = CT_max_ of host caterpillar, b = CT_max_ of parasitoid wasp, c = CT_max_ of hyperparasitoid wasp.

The maximum daily air temperature recorded near the study site in the past 124 years was 42°C, which was observed twice (Fig 3). The total number of days observed at or above 40°C was 34 (0.03%; Fig 3).

## Discussion

To our knowledge, this is the first study to test for differences in the upper thermal tolerance of component species in a natural H-P-HP system. Based on our estimates of CT_max_, the upper limits to performance differ by several degrees between the caterpillar, *M*. *sexta*, and adults of its major parasitoid, *C*. *congregata*, and between *C*. *congregata* and adults of one of its major hyperparasitoids, *Conura* sp. The specific average values of CT_max_ that we estimated for the component species ranged from 42–48°C, which is well within the range of previously measured values for insects [54].

Only two other studies that we are aware of have made similar comparisons in H-P-HP systems, both of which focused on cold tolerance. Campbell et al. [55] estimated lower developmental threshold temperatures in several aphid species and their parasitoids, including a H-P-HP system. In general, they found that adult parasitoids and hyperparasitoids had similar or slightly higher threshold temperatures than their hosts. Rice and Allen [56] estimated lower developmental threshold temperatures in a chrysomelid beetle, three of its parasitoids, and a hyperparasitoid of one of the parasitoids. Thresholds differed by less than 0.5*°*C among the parasitoid species but were about 2°C higher in the host and about 4°C higher in the hyperparasitoid. These quantitative differences in lower thermal tolerance among component H-P-HP species are comparable to the differences we found in upper thermal tolerance in our system.

Numerous other studies have compared various aspects of thermal performance between parasitoids and their hosts (i.e., between the first two trophic levels). Nealis et al. [57] studied lower developmental threshold temperatures in the non-native butterfly *Pieres rapae* from Canada and Australia, and three parasitoids from its native range that were introduced for biocontrol. In general, immature stages of parasitoids had similar or slightly higher threshold temperatures for development than their host. As in our study, van Baaren et al. [7] found the aphid, *Sitobion avenae*, had an upper thermal tolerance about 1–2°C higher than adults of three parasitoid wasp species. Hughes et al. [58] found adult wasps had both higher heat tolerance and lower cold tolerance than their aphid host; although, the data derived from mass-reared laboratory populations which may not be representative of wild populations (e.g. [59, 60]). Wang et al. [61] compared entire thermal performance curves of a fruit fly introduced to California and two wasp parasitoids from its native range in Africa. One parasitoid had lower heat tolerance than the host, whereas the other had higher heat tolerance. Finally, Bahar et al. [62, 63] found several differences between the introduced caterpillar, *Plutella xylostella*, and its North American parasitoid, *Diadegma insulare*, that suggest larvae of this parasitoid species have lower heat tolerance than their recently acquired host. Beyond parasitoids, other studies involving more traditional host-parasite systems have shown similar differences in various aspects of thermal performance [64–67].

In addition to variation among species, we found significant variation in upper thermal tolerance within the host, *M*. *sexta*, with respect to both instar and parasitism. Evidence for ontogenetic variation in thermal tolerance is widespread in insects [24–27]. We found that older and much larger 5^th^ instar *M*. *sexta* had about 1.5°C lower CT_max_ than 3^rd^ and 4^th^ instars. This result is consistent with a prior study suggesting that thermal tolerance decreases through larval ontogeny in *M*. *sexta*, with 5^th^ instars being more sensitive to high temperatures than earlier instars ([68], and see [69]). We also found that caterpillars parasitized by *C*. *congregata* had about 1.0°C lower CT_max_ than non-parasitized caterpillars. To our knowledge, this is the first study to show a difference in upper thermal tolerance between parasitized and non-parasitized hosts in a host-parasitoid system. Müller and Schmid-Hempel [70] demonstrated that parasitized bumblebees selected cooler climates by staying outside the nest overnight, which slowed development and reduced the success of its fly parasitoid. More generally, it is well-known that parasites and pathogens can modify host thermal tolerance (e.g. [28–32]) and host thermoregulatory behavior (e.g. [30, 31, 71, 72]).

Taken together, these results imply that significant differences in thermal performance among and within component species in host-parasite systems are common in nature. Although the specific reasons for the differences are unknown, they are presumably related to the evolutionary histories of habitat use and associated thermal environments and biophysical properties of the component species. Regardless of the reasons, these differences suggest that species may often respond differently and in complex ways to changes in thermal regimes and that large-scale climate change is likely to disrupt many existing interactions to some degree. In fact, climate change has been a driver of change in host-parasite systems throughout earth history [12, 14, 17, 73]. The current emerging infectious disease (EID) crisis is but one example [2, 18, 20, 73]. EIDs arise when parasites and pathogens encounter new, evolutionarily novel hosts. Large-scale perturbations like climate change are associated with shifting host and parasite phenologies, abundances and distributions, which increase biotic mixing, which is the source of EIDs [2, 18, 20, 73].

### Relevance of CT_max_ to organisms in nature

Several simple indices to assess the vulnerability of organisms to climate change have been proposed, including “warming tolerance”, sometimes defined as the difference between CT_max_ and the average habitat temperature [54, 74–76], and “thermal safety margin” or “thermal buffer”, sometimes defined as the difference between CT_max_ and the maximal habitat temperature [77–79]. Whether such simple measures can capture enough inherent complexity of the problem to be useful remains to be seen. In the meantime, such proposals draw attention to the extreme limits to thermal performance and bring into question their relevance to organisms in nature. For instance, how often do organisms experience temperatures near or above their upper thermal limits? Although it may be infrequent, even occasional exposure to extreme high temperatures can have large impacts on the performance, abundance, and distribution of ectotherms [6, 11, 78, 80–85].

Ideally, information to gauge the frequency of exposure of small ectotherms to extreme high temperatures would derive from detailed studies of the microclimates and body temperatures they experience in their natural habitats [86–88]. In the absence of such information, we used maximum air temperatures recorded near the study site in the past 124 years as a crude but potentially informative tool. The highest daily air temperature recorded near the study site was 42°C, which occurred twice, and the frequency of days with temperatures at or above 40°C was less than a tenth of a percent. This is consistent with other data summarized for sites even closer to our study site for the period 1949–2001, when the highest recorded air temperature was 40°C [89]. Two species (*M*. *sexta*, *Conura* sp.) we studied had average CT_max_ values that were several degrees higher than 42°C while one species (*C*. *congregata*) had a value that was equal to 42°C. Thus, maximal air temperatures recorded near the study site have never exceeded the CT_max_ of the three species in our study in the past 124 years. On the other hand, given the coarseness of the data, the fact that our estimated CT_max_ values were near the highest ever recorded maximum air temperatures suggests the organisms may occasionally experience body temperatures near their upper thermal limits in the field. Furthermore, climate projections predict an increase in heat waves and extreme high temperatures in the next 50–100 years in many temperate regions [90, 91]. Some models suggest this may have an especially large impact on mid-latitude species [78, 81], such as those studied here. Taken at face value, our results suggest that the parasitoid *C*. *congregata* may be the most vulnerable species in this H-P-HP system to the predicted warming because it currently has the lowest upper thermal tolerance.

Of course, organisms will begin to experience the sub-lethal effects of high temperature before reaching the extreme limits to thermal performance captured by measures such as CT_max_. We showed recently evidence that the efficiency of mitochondria in harnessing oxygen and organic substrates into cellular energy (ATP) drops off rapidly past 35°C in a laboratory strain of *M*. *sexta* [37]. This drop in mitochondrial efficiency is correlated with decreased larval growth rates and increased metabolic rates [36], suggesting that the “cost of living” goes up dramatically for caterpillars past 35°C, long before reaching the estimated CT_max_ of 46°C. The data in Fig 3 and those reported by Tilson et al. [89] suggest *M*. *sexta* may routinely experience temperatures near and above 35°C in our study area over the course of a typical summer. In southern California, *M*. *sexta* caterpillars frequently experienced body temperatures above 36°C during the day in July [92]. While body temperatures may rarely approach or exceed the extreme limits to performance, selection on performance at less extreme temperatures may indirectly drive their evolution and partly account for differences in CT_max_ among and within species.

### Methodological issues with measuring CT_max_

There are three issues with our methodology for estimating CT_max_ that are important to note. First, different ramping rates for the same species can give different values of CT_max_ and the same ramping rate can affect species differently [49–51]. Second, thermal history, which was unknown for our field-collected organisms, can affect CT_max_ [93]. Third, there is a question of how comparable CT_max_—as measured by the onset of muscular spasms [46, 47]- is between organisms as different as wasps and caterpillars. We recorded videos of each individual, which allowed us to observe the temperature at the first observable muscular spasm very precisely. Were the spasms we observed representative of the same “event” in soft-bodied caterpillars and hard-bodied wasps? One way to address this question would be to use other measures of thermal tolerance (e.g., knockdown temperature, lethal temperature) to independently corroborate findings based on muscular spasms. Lighton and Turner’s [48] method of thermolimit respirometry, which measures CT_max_ based on patterns of respiration as opposed to observing behavior, could be an especially useful technique to compare among different types and sizes of organisms. Due to limited samples of field-collected organisms, we were unable to address these issues in our study; however, it would be highly interesting to repeat our experiment using different ramping rates, thermal histories, and methods of estimating thermal tolerance to examine how methodology may affect the results.

### Conclusions

Our study demonstrates differences in upper thermal limits among and within species in a natural tritrophic system involving parasitoids and their hosts. Other studies demonstrate similar differences in various other aspects of thermal performance between hosts and parasites. Such differences imply that climate change will be largely disruptive to these systems and that their responses will be highly varied and complex.

## Supporting information

S1 Appendix Data set analyzed in this study. (XLSX)

S2 Appendix Calibration of CT_max_ measurements. (DOCX)
