# Supplementary material for: Upper thermal limits differ among and within component species in a tritrophic host-parasitoid-hyperparasitoid system
Source: PLoS One. 2018 Jun 12;13(6):e0198803. doi: 10.1371/journal.pone.0198803 (PMC5997305; doi:10.1371/journal.pone.0198803)
Supplement: S2 Appendix — (DOCX) [file pone.0198803.s002.docx]

**Supplementary Information – S2 Appendix – Calibration of CT_max_ Measurements**

Upper thermal limits differ among and within component species in a tritrophic host-parasitoid-hyperparasitoid system

Salvatore J. Agosta, Kanchan A. Joshi, Karen M. Kester

*Corresponding author: [sagosta@vcu.edu](mailto:sagosta@vcu.edu)

Direct measurements of CT_max_ were made based on the temperature of the water bath. To calibrate these measurements to better reflect the body temperatures of the organisms, we conducted two separate trials using a 4^th^ and 5^th^ instar *Manduca sexta*. These organisms represented the two largest size classes in our experiment. Calibration was done by simultaneously measuring the water bath temperature (T_water_; green symbols), air temperature inside an empty glass vial (T_air_; red symbols), and body temperature of a dead (frozen-then-thawed) caterpillar (T_body_; blue symbols) every two minutes during a thermal ramp at 0.25°C min^-1^ (i.e., the same rate used during CT_max_ measurements for the experiment). These data were used to test the assumption that T_water_ = T_air_ = T_body_. To the extent that T_water_ ≠ T_air_ ≠ T_body_, the data were then used to calculate correction factors for CT_max_ based on T_body_. See article for further details.
